# Time-restricted eating and gut microbiota alpha diversity in adults with overweight or obesity: an updated rapid systematic review

**DOI:** 10.3389/fnut.2026.1910444

**Published:** 2026-09-09

**Authors:** Yibing Melody Zhai, Xinyu Liu, Zeyou Wang, Min Wang, Guangbin Tang, Yijie Liu, Hao Liang

**Affiliations:** 1 Department of Laboratory Medicine, The Second Xiangya Hospital, Central South University, Changsha, Hunan, China; 2 Department of Oncology, Research Center of Cancer Diagnosis and Therapy, The First Affiliated Hospital of Jinan University, Guangzhou, China; 3 Research Center for Life Philosophy, Hunan Academy of Social Sciences, Changsha, Hunan, China

**Keywords:** alpha diversity, GRADE, gut microbiota, obesity, systematic review, time-restricted eating

## Abstract

**Background:**

Time-restricted eating (TRE) is widely used for weight management, but its effect on gut microbiota alpha diversity in adults with overweight or obesity remains uncertain.

**Methods:**

We updated a rapid systematic review of intervention studies in adults with body mass index (BMI) of at least 25 kg/m^2^. The original searches covered PubMed, Web of Science Core Collection, Embase, and CENTRAL from inception to 10 February 2026. For the update period from 11 February through 4 August 2026, PubMed was rerun and Europe PMC, OpenAlex, and ClinicalTrials.gov were searched as supplementary discovery sources, with backward and forward citation checking. Eligible studies applied a daily eating window of 12 h or less for at least 2 weeks and reported an alpha-diversity metric. Risk of bias was assessed with RoB 2, ROBINS-I, or the JBI quasi-experimental checklist, as appropriate. Certainty was assessed with GRADE. Because study designs, comparators, metrics, and reporting were not sufficiently compatible, results were synthesized narratively and no meta-analysis was performed.

**Results:**

Nine reports included 517 participants with microbiome data (14–138 per study), including five randomized comparative reports (*n* = 377), two non-randomized controlled studies, and two uncontrolled longitudinal analyses. Three randomized reports found no clear between-group change, one reported timing-dependent divergence between early and late TRE, and one reported increased richness measures after 6-h TRE. Non-randomized and uncontrolled reports generally found no clear change, but numerical estimates were frequently unavailable. The certainty of the overall evidence was very low because of risk of bias, inconsistency, imprecision, and incomplete outcome reporting.

**Conclusion:**

Available evidence does not establish either a beneficial effect or an absence of effect of TRE on gut microbiota alpha diversity. Timing-specific differences are plausible but remain hypothesis-generating. Adequately powered trials should prespecify microbiome outcomes, retain early and late TRE as separate interventions, and report paired and between-group estimates with uncertainty.

## Introduction

Time-restricted eating (TRE) confines daily energy intake to a recurring eating window, commonly 6–12 h, without necessarily prescribing what or how much participants eat. Randomized and pragmatic trials suggest that TRE can reduce body weight and improve selected cardiometabolic markers in some populations, although effects vary with energy intake, adherence, and the timing of the eating window (1–3).

The gut microbiome participates in energy harvest, immune regulation, and host metabolism (4). Alpha diversity summarizes richness and evenness within a microbial community. Lower diversity has been associated with obesity-related phenotypes, but alpha diversity is a nonspecific ecological descriptor rather than a direct measure of microbial function or health (5). Feeding-fasting cycles also contribute to diurnal microbial oscillations, creating a biologically plausible link between meal timing and microbiome structure (6).

Previous reviews combined TRE with alternate-day fasting, 5:2 diets, Ramadan fasting, and prolonged fasting, and included heterogeneous populations (7). A focused synthesis in adults with overweight or obesity could therefore be useful. However, strict eligibility criteria also produce a small and methodologically diverse evidence base. The original submission attempted to pool two studies, but re-extraction showed that the studies differed in design and intervention structure and did not supply the information needed for a defensible common effect estimate.

We therefore updated the search, reverified eligibility and microbiome sample sizes, retained multi-arm interventions as separate schedules, and replaced the previous meta-analysis with a structured narrative synthesis. The objective was to describe the direction and certainty of the evidence on TRE and gut microbiota alpha diversity in adults with overweight or obesity, without interpreting statistical non-significance as proof of no effect.

## Methods

### Protocol

This rapid systematic review was reported in accordance with PRISMA 2020 (8). The protocol was not prospectively registered; therefore, no registration number is available. The review question, eligibility criteria, and original database searches were established before screening. The decision not to pool was made after the revision-stage audit identified incompatible designs, inappropriate multi-arm combination, and insufficient paired data. This deviation is reported transparently.

### Inclusion and exclusion criteria

According to the PICOS framework, eligible populations were adults aged at least 18 years with overweight or obesity, operationalized as BMI of at least 25 kg/m^2^. Eligible interventions used a recurring TRE window of 12 h or less for at least 2 weeks. Comparators were unrestricted eating, an otherwise similar dietary program, another TRE schedule, or baseline for eligible uncontrolled intervention studies. Eligible outcomes were gut microbiota alpha-diversity metrics, including Shannon, Simpson, richness or observed features, Chao1, and evenness. Randomized, crossover, non-randomized controlled, and prospective before-after intervention studies were eligible.

Studies were excluded if they evaluated Ramadan fasting, alternate-day fasting, 5:2 diets, prolonged multi-day fasting, animals, protocols without results, or multicomponent interventions without an isolatable TRE arm. Reports without an eligible overweight or obesity population, without an alpha-diversity outcome, or without sufficient intervention-specific information were also excluded.

BMI was retained as the adiposity criterion because it was prespecified, consistently reported across candidate studies, and permitted a reproducible threshold across settings. Waist circumference, waist-to-hip ratio, and waist-to-height ratio identify central adiposity and add clinically relevant information (9), but were not uniformly reported or used for trial eligibility. Central-adiposity criteria or measurements were extracted when reported; reliance on BMI alone is acknowledged as a limitation.

### Information sources and search

The original search covered PubMed, Web of Science Core Collection, Embase via Ovid, and Cochrane CENTRAL from inception to 10 February 2026. For the update period from 11 February through 4 August 2026, we reran PubMed, searched Europe PMC and OpenAlex as supplementary discovery sources, searched ClinicalTrials.gov as a study registry, and performed backward and forward citation checking of included reports and relevant reviews. Europe PMC provides life-sciences literature, whereas OpenAlex is an open multidisciplinary scholarly index. Overlap among sources was removed during deduplication. Complete source-specific strategies, dates, and record counts are provided in Supplementary Table S1.

### Study selection and data extraction

Two reviewers independently screened titles and abstracts and then full texts. At revision, data were re-extracted for study design, randomization, microbiome-specific analytic sample, paired sample availability, population, adiposity criteria, eating-window schedule, comparator, co-interventions, duration, sequencing platform, alpha-diversity metric, analysis method, estimates, and exact *p* values when reported. Disagreements were resolved by discussion. Randomized enrollment, trial completion, microbiome sampling, and paired microbiome analysis were distinguished to avoid using parent-trial enrollment as the outcome sample.

### Risk of bias

Randomized comparative reports were assessed with RoB 2 (10). Non-randomized controlled studies were assessed with ROBINS-I (11). Uncontrolled longitudinal studies were appraised with the JBI quasi-experimental checklist because a randomized relative-effect estimate was not available (12). Judgments were based on the microbiome alpha-diversity outcome rather than the parent trial’s primary clinical outcome. Tool-specific results are presented separately and were not converted to a common set of domains.

### Certainty of evidence

GRADE was used to assess randomized comparative evidence, non-randomized or uncontrolled evidence, and the overall conclusion for alpha diversity (13). The certainty of evidence was categorized as high, moderate, low, or very low. Ratings considered risk of bias, inconsistency, indirectness, imprecision, and publication bias for each defined evidence body.

### Synthesis

Because the included studies differed in design, comparator, eating-window schedule, alpha-diversity metric, and outcome reporting, we used a structured narrative synthesis following the Synthesis Without Meta-analysis (SWiM) reporting guideline (14). Studies were grouped by design and alpha-diversity metric, and author-reported estimates, *p* values, and directions of change were summarized in text and tables. Early and late TRE arms were kept separate, and repeated-measures findings were reported as analyzed by the original authors. No meta-analysis or sensitivity analysis was conducted.

## Results

### Study selection

**Figure 1:**
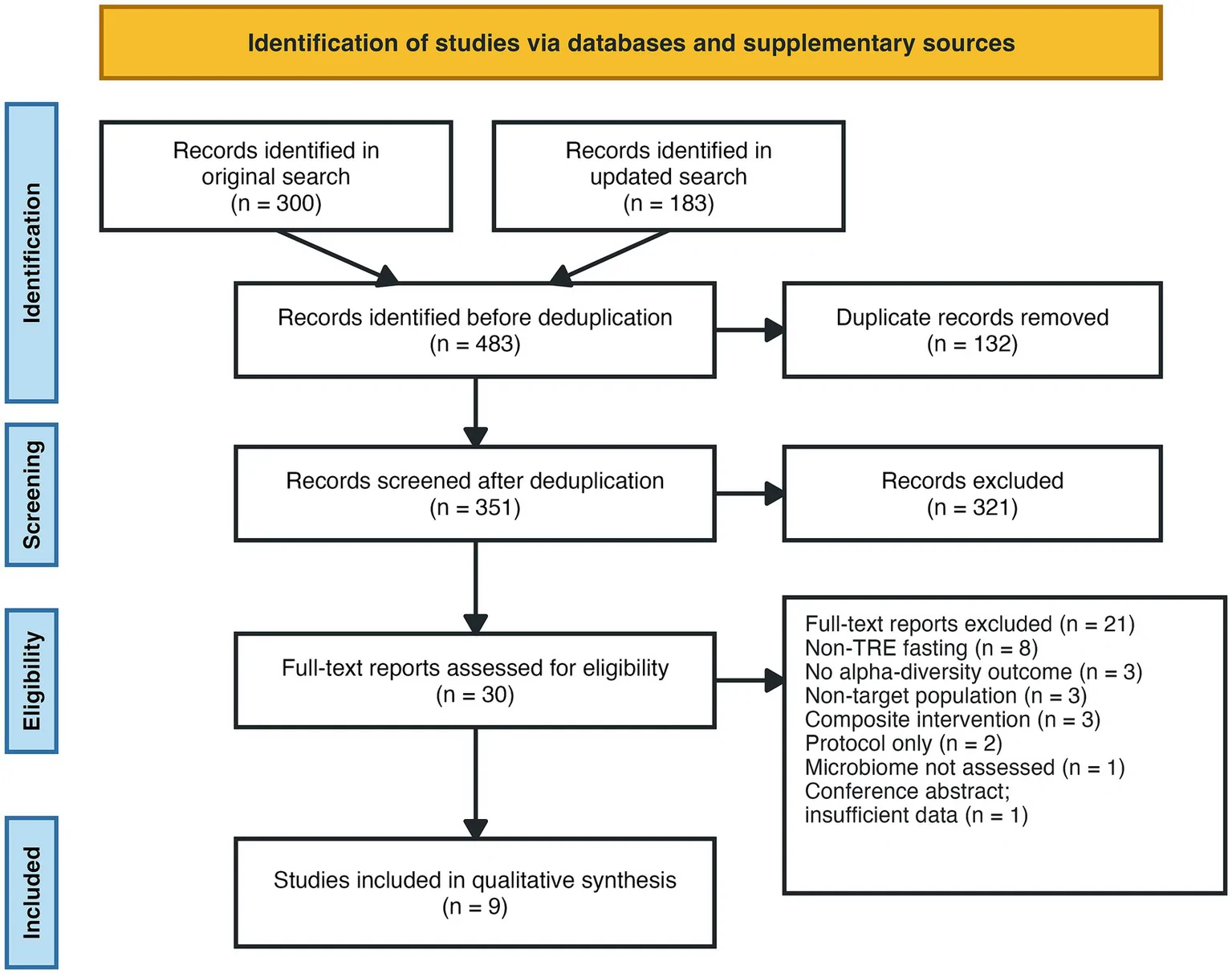
PRISMA 2020 flow diagram. The original search and update sources yielded 483 records; 351 unique records were screened and nine studies were included.

The original and update searches identified 483 records. After 132 duplicates were removed, 351 unique records were screened and 30 full-text reports were assessed. Twenty-one full-text reports were excluded, leaving nine eligible studies (Figure 1). The update search added Zhang et al. and Dote-Montero et al. (15, 16).

### Study and sample characteristics

The nine reports contributed 517 participants with microbiome data, reconstructed as 14, 49, 26, 51, 87, 16, 76, 60, and 138 participants across the individual studies (15–23). Five randomized comparative reports contributed 377 participants; two non-randomized controlled and two uncontrolled reports contributed 140. The 517-participant count refers to microbiome analytic samples, not parent-trial enrollment. Johnson et al. included 21 samples from 16 participants, but only five participants supplied paired baseline and follow-up stools (22). Li et al. randomized 96 participants, 88 completed the 12-week intervention, and paired sequencing succeeded for 87 (TRE main effect, 43 versus 44) (21). Dote-Montero et al. randomized 197 participants, but 138 had analyzable paired microbiome data (16).

### Interventions and outcome measurement

**Figure 2:**
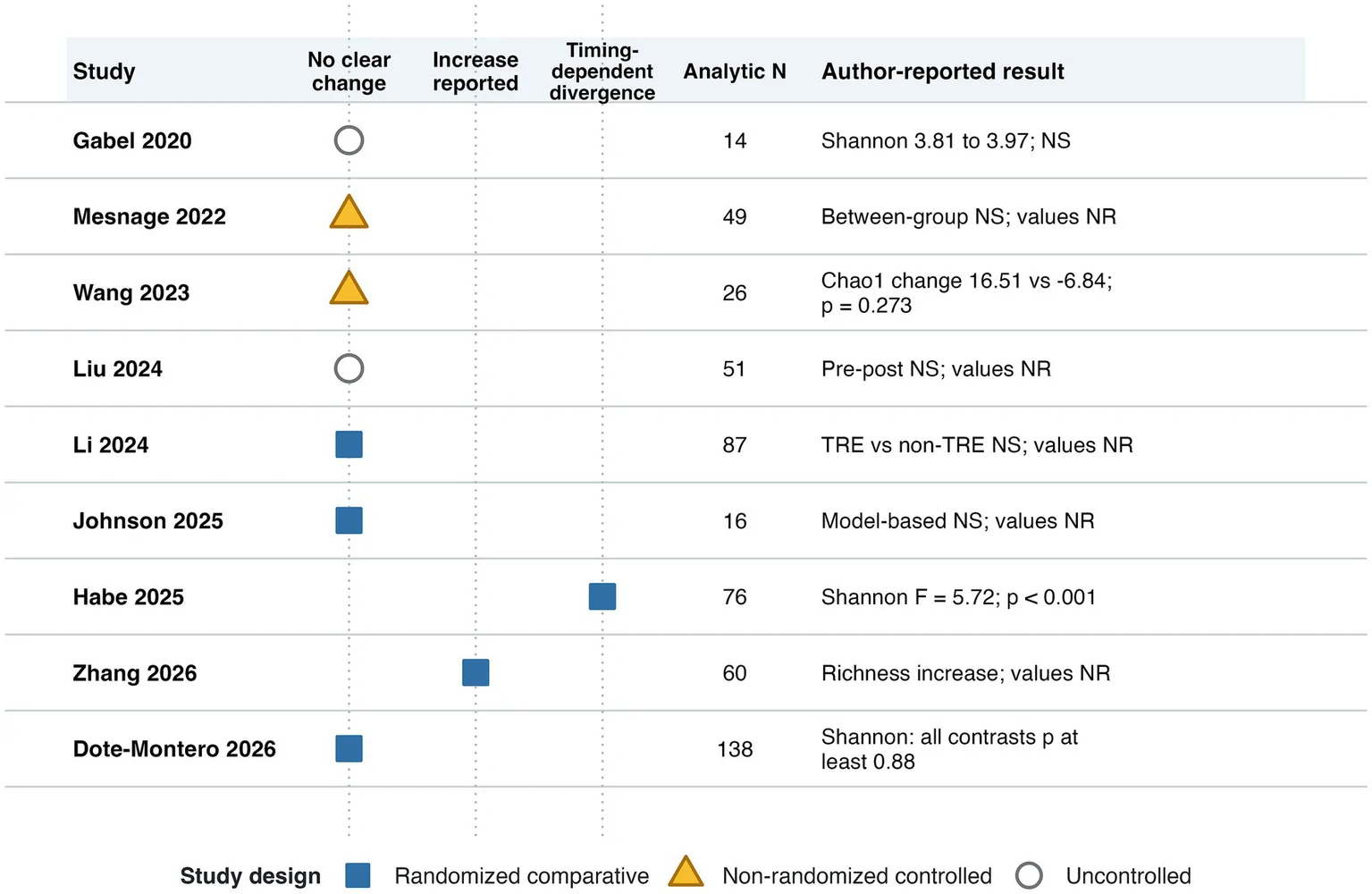
Study-level effect-direction plot. Symbol position indicates the author-reported direction or pattern, not a common effect magnitude; symbol shape and color indicate study design. Early and late TRE were retained as separate schedules. NR, not reported; NS, not statistically significant.

Eating windows ranged from 6 to less than 12 h, and interventions lasted 8 weeks to 12 months. Several studies combined TRE with energy restriction or dietary education, whereas Gabel, Johnson, and Zhang used ad libitum TRE prescriptions. Eight reports used 16S rRNA amplicon sequencing or shotgun metagenomics; Johnson and Li used shotgun metagenomics. Alpha-diversity metrics included Shannon, Simpson, Chao1, observed features or richness, number of taxa, and Pielou evenness (Table 1; Figure 2).

**Table 1:** Characteristics and *α*-diversity findings of included studies.

Study and design	Sample and population	TRE and comparator	Alpha-diversity finding
Gabel 2020 Uncontrolled prospective pre-post	*n* = 14; Adults with obesity. BMI about 35 kg/m^2^; obesity required	8-h TRE; no concurrent comparator; 10:00–18:00; 12 weeks. Ad libitum prescription; spontaneous energy intake reduction	Shannon; OTU richness: Shannon 3.81 ± 0.40 at baseline, 3.88 ± 0.41 at week 1, and 3.97 ± 0.41 at week 12; not statistically significant
Mesnage 2022 Prospective non-randomized controlled	*n* = 49; Adults with obesity. BMI 30–45 kg/m^2^; waist circumference measured	TRE *n* = 25 versus time-unrestricted eating *n* = 24; <12 h and last meal before 19:00; 12 weeks. Same 500–1,000 kcal/day deficit in both groups	Alpha diversity not otherwise specified: No significant between-group difference in alpha diversity; numerical values and exact *p* value not reported (data not shown)
Wang 2023 Prospective non-randomized controlled pilot	*n* = 26; Adults with overweight/obesity and CKD stages 3–4. BMI at least 24 kg/m^2^; mean BMI about 28–29 kg/m^2^	TRF *n* = 13 versus conventional diet *n* = 14 among completers; 8 h; start between 07:00 and 12:00; 12 weeks. Low-protein diet in both groups; intake decreased	Chao1: Change 16.51 ± 64.94 in TRF versus −6.84 ± 41.62 in control; *p* = 0.273
Liu 2024 Uncontrolled longitudinal subgroup of an RCT	*n* = 51; Adults with obesity. BMI 28–45 kg/m^2^	TRE-assigned subgroup only; participants categorized by weight-loss response; 08:00–16:00; 12 months. Calorie restriction in all analyzed participants	Shannon; Simpson; richness: No significant pre-post change in Shannon, Simpson, or richness; numerical values and exact *p* values not reported
Li 2024 2 × 2 factorial randomized controlled feeding trial	*n* = 87; Adults with overweight/obesity. BMI 24–35 kg/m^2^	TRE main effect *n* = 43 versus non-TRE *n* = 44 across healthy low-carbohydrate factor; 10 h; 12 weeks. About 25% caloric restriction in all four cells	Shannon: No significant within-group shift or TRE-versus-non-TRE difference; exact *p* value not reported
Johnson 2025 Randomized controlled trial secondary microbiome analysis	*n* = 16; Adults with overweight/obesity. BMI at least 25 kg/m^2^; microbiome subset median BMI about 30–32 kg/m^2^	TRE *n* = 8 versus non-TRE *n* = 8 with at least one stool sample; Self-selected 8 h; 12 weeks. Ad libitum	Shannon; Simpson; number of taxa: No treatment-by-time effect in linear mixed models; exact metric-specific *p* values not reported
Habe 2025 Three-arm randomized controlled trial secondary microbiome analysis	*n* = 76; Adults with overweight/obesity. BMI 25–35 kg/m^2^ and metabolic abnormality	Early TRE + ER *n* = 33; late TRE + ER *n* = 23; ER alone *n* = 20; 08:00–16:00 or 12:00–20:00; 12 weeks. Energy restriction in all arms	Shannon; Simpson; richness: Adjusted changes differed across the three arms: Shannon *F* = 5.72, *p* < 0.001; Simpson *F* = 6.72, p < 0.001; richness *F* = 3.99, *p* = 0.01. Late TRE showed the largest decrease, early TRE was stable, and ER increased at 12 weeks
Zhang 2026 Three-arm randomized controlled trial	*n* = 60; Young adults with overweight/obesity. BMI at least 24 kg/m^2^	Early TRE; late TRE; ad-libitum control; 07:00–13:00 or 12:00–18:00; 8 weeks. Ad libitum	Genus-level Chao1; observed species: The report states that both 6-hour TRE schedules increased alpha diversity. Panel E of Figure S2 in the Supporting Information published with Zhang et al. (22) displays the Chao1 and observed-species results, but arm-specific numerical estimates and exact between-group *p* values are not reported
Dote-Montero 2026 Four-arm multicenter randomized controlled trial secondary analysis	*n* = 138; Adults with overweight/obesity. BMI 25–40 kg/m^2^ plus abdominal obesity (waist at least 95 cm men or 82 cm women)	Usual care *n* = 35; early TRE *n* = 36; late TRE *n* = 36; self-selected TRE *n* = 31 in paired microbiome analysis; 8 h; 12 weeks. Mediterranean-diet education in all arms	Shannon; Simpson; observed features; Pielou evenness: No between-group differences in changes: Shannon all *p* at least 0.88; Simpson at least 0.50; observed features at least 0.49; Pielou at least 0.78

BMI, body mass index; CKD, chronic kidney disease; ER, energy restriction; NR, not reported; NS, not statistically significant; RCT, randomized controlled trial; TRE, time-restricted eating.

### Randomized comparative evidence

Li et al. found no significant within-group shift in Shannon diversity and no TRE-versus-non-TRE difference; exact numerical estimates and *p* values were not reported (21). Johnson et al. found no treatment-by-time effect for Shannon, Simpson, or number of taxa in mixed models using 21 samples from 16 participants; metric-specific *p* values were not reported, and only five participants had paired samples (22). Dote-Montero et al. found no difference among usual care, early TRE, late TRE, and self-selected TRE in change in Shannon (all comparisons *p* ≥ 0.88), Simpson (*p* ≥ 0.50), observed features (*p* ≥ 0.49), or Pielou evenness (*p* ≥ 0.78) among 138 participants with paired microbiome data (16).

### Timing-dependent and richness findings

Habe et al. retained early TRE plus energy restriction (*n* = 33), late TRE plus energy restriction (*n* = 23), and energy restriction alone (*n* = 20) as separate arms. Changes differed across groups for Shannon (*F* = 5.72, *p* < 0.001), Simpson (*F* = 6.72, *p* < 0.001), and richness (*F* = 3.99, *p* = 0.01). Late TRE showed the largest reduction, early TRE remained comparatively stable, and energy restriction alone increased diversity at 12 weeks (23). Zhang et al. randomized 60 participants to 6-h early TRE, 6-h late TRE, or an ad-libitum control and reported increases in genus-level Chao1 and observed-species measures after TRE; accessible text and supplementary captions did not provide arm-specific numerical estimates or exact comparative *p* values (15). Certainty in the randomized comparative evidence was graded very low because of risk of bias, discordant findings, small effective samples, imprecision, and incomplete outcome reporting (Supplementary Table S4).

### Non-randomized and uncontrolled evidence

Gabel et al. reported Shannon values of 3.81 ± 0.40 at baseline, 3.88 ± 0.41 at week 1, and 3.97 ± 0.41 at week 12 in 14 adults with obesity; the change was not statistically significant (17). Mesnage et al. reported no significant between-group difference in alpha diversity between TRE (*n* = 25) and time-unrestricted eating (*n* = 24), but numerical values and the exact *p* value were not reported (‘data not shown’) (18). Wang et al. reported a Chao1 change of 16.51 ± 64.94 with TRE versus −6.84 ± 41.62 with the conventional diet (*p* = 0.273) among 26 participants with paired microbiome data (19). Liu et al. analyzed only 51 of 69 participants assigned to TRE in the parent TREATY trial and reported no pre-post difference in Shannon, Simpson, or richness over 12 months; this was not a randomized TRE-versus-control microbiome comparison (20).

### Risk of bias

**Figure 3:**
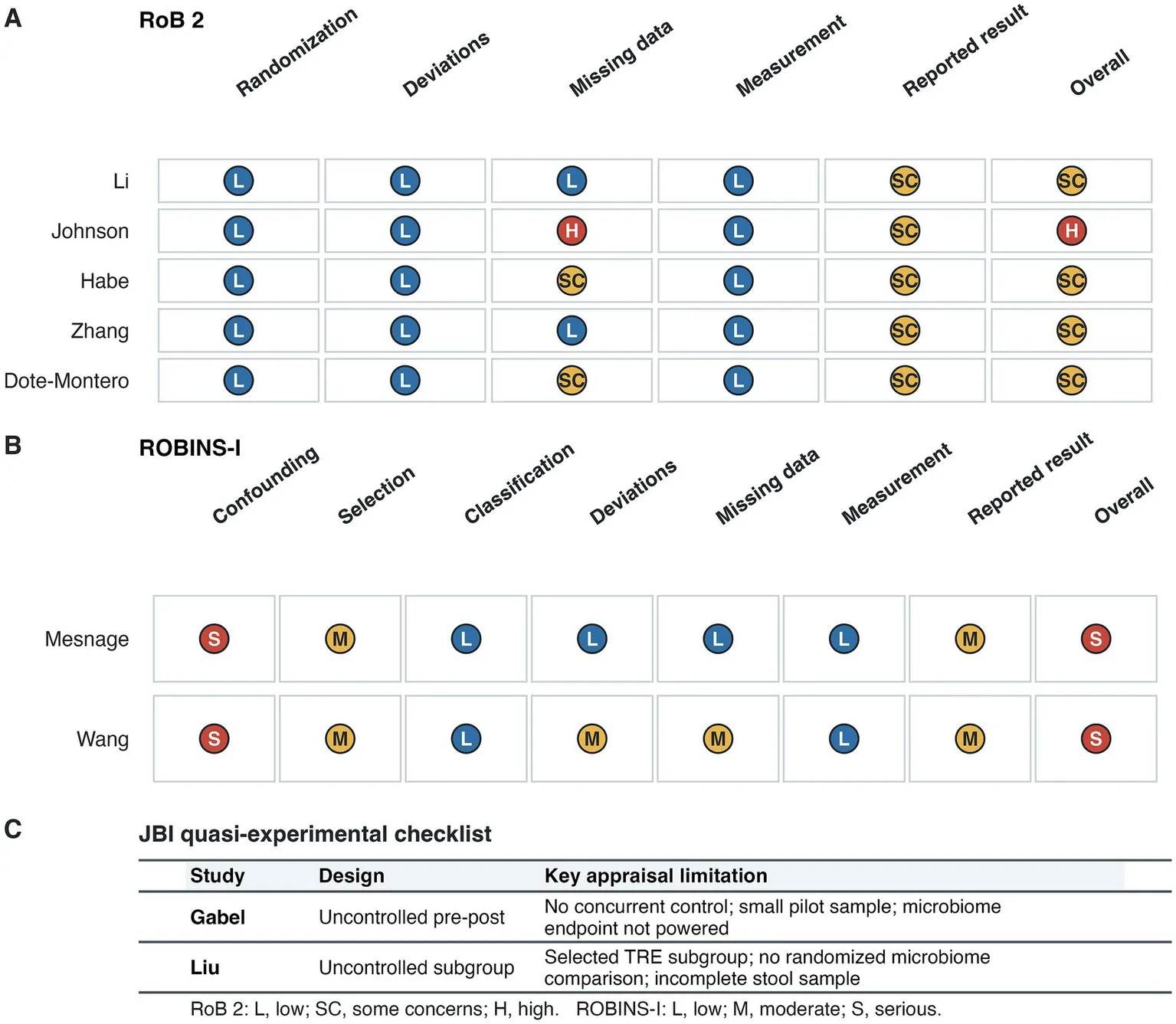
Tool-specific risk-of-bias assessment. **(A)** Presents RoB 2 judgments for randomized comparative studies; **(B)** Presents ROBINS-I judgments for non-randomized controlled studies; **(C)** Summarizes key JBI appraisal limitations for uncontrolled studies. Domains and ratings are not mapped across tools.

Among RoB 2 assessments, Li, Habe, Zhang, and Dote-Montero had some concerns, mainly because alpha diversity was a secondary outcome or reporting was incomplete; Johnson was at high risk because paired outcome data were available for only five participants. In the separate ROBINS-I assessment, Mesnage and Wang were at serious risk, primarily because preference-based allocation created substantial confounding. The JBI appraisal of Gabel and Liu identified major interpretive limitations from the absence of a concurrent microbiome comparison and, for Liu, use of a selected TRE subgroup. Because the three tools have different domains and purposes, their ratings are displayed in separate panels and should not be compared as a common scale (Figure 3; Supplementary Table S3).

## Discussion

After re-extraction and an updated search, the evidence base comprised nine reports and 517 microbiome participants. The central conclusion is uncertainty: current studies do not establish a consistent beneficial effect of TRE on alpha diversity, but they also do not establish that TRE has no effect. Statistical non-significance in small or incompletely reported studies is compatible with both small effects and important effects that the studies could not resolve.

The original meta-analysis was not retained because the studies did not estimate a sufficiently comparable effect. Li was a 2 × 2 factorial trial, Habe was a three-arm timing trial with different early and late TRE schedules, and Gabel was an uncontrolled before-after study. Combining these designs would obscure clinically important differences and could produce a misleading summary. The revised review therefore reports the study-level findings in a structured narrative synthesis. Because no meta-analysis was performed, heterogeneity statistics and sensitivity analyses were not applicable.

The contrast between Habe and Zhang, on the one hand, and the remaining comparative studies, on the other, suggests that eating-window timing, window duration, energy restriction, and outcome metric may modify results. Habe reported divergence across early and late 8-h windows, whereas Zhang reported increases in richness after 6-h schedules. Dote-Montero, the largest microbiome analysis, found no schedule-specific difference in Shannon or related indices. These observations make timing a reasonable hypothesis for future trials, not a basis for clinical prescription.

Alpha diversity is an incomplete surrogate for microbiome function. A stable Shannon index does not rule out changes in specific taxa, gene pathways, metabolites, or diurnal oscillation. Conversely, increased richness is not automatically beneficial. The included evidence therefore cannot determine whether microbiome changes mediate weight loss or other metabolic outcomes of TRE. Previous mediation and bile-acid statements were removed because the reviewed alpha-diversity data do not test those mechanisms.

We used BMI to maintain a reproducible eligibility criterion across all studies. This decision can miss adults with central adiposity at a lower BMI and cannot distinguish visceral from subcutaneous fat. Waist circumference and related indices should be reported in future trials and considered in prespecified subgroup analyses (9). The Dote-Montero trial is informative in this respect because it required both overweight or obesity by BMI and abdominal obesity by waist circumference.

This review has limitations. It used a rapid-review workflow, and the protocol was not prospectively registered. The update used open bibliographic and registry sources rather than rerunning every subscription database through 4 August 2026. Outcome reporting was often incomplete, and several microbiome analyses were secondary or included selected subsets. Study populations, co-interventions, eating windows, sequencing methods, taxonomic levels, and alpha-diversity metrics varied. Conference-only evidence with insufficient arm-specific data was excluded. Publication bias could not be evaluated statistically with so few heterogeneous studies.

Future trials should prespecify microbiome outcomes and analysis plans, report randomized, sequenced, and paired sample counts separately, analyze change with appropriate repeated-measures models, and provide arm-specific estimates with confidence intervals. Multi-arm trials should preserve early, late, and self-selected schedules. Standardized sequencing, reporting of Shannon and richness metrics, and parallel functional measurements would make subsequent synthesis more informative.

## Conclusion

The certainty of evidence on TRE and gut microbiota alpha diversity in adults with overweight or obesity is very low. Available studies neither demonstrate a consistent benefit nor justify a conclusion of no effect. Timing-dependent differences have been reported, but require confirmation in adequately powered, prespecified trials with complete paired and between-group reporting.

## Data Availability

The original contributions presented in the study are included in the article/Supplementary material, further inquiries can be directed to the corresponding author.
