# Supplementary material for: Time-restricted eating and gut microbiota alpha diversity in adults with overweight or obesity: an updated rapid systematic review
Source: Front Nutr. 2026 Sep 9;13:1910444. doi: 10.3389/fnut.2026.1910444 (PMC13597893; doi:10.3389/fnut.2026.1910444)
Supplement: Supplementary file 1 [file Table_1.docx]

# Supplementary Table S1. Search Strategies

Original database searches were conducted on 10 February 2026. PubMed was rerun for the update period from 11 February through 4 August 2026; Europe PMC and OpenAlex were used as supplementary discovery sources, and ClinicalTrials.gov was searched as a study registry. Counts are records returned before cross-source deduplication. Search syntax was adapted to each source.

| **Source** | **Search date / coverage** | **Records** | **Search strategy** |
| --- | --- | --- | --- |
| **PubMed (original)** | Searched 10 February 2026; inception to search date | 176 | ("time-restricted eating" OR "time restricted eating" OR "time-restricted feeding" OR "time restricted feeding" OR TRE OR TRF) AND ("gut microbiota" OR "gut microbiome" OR "intestinal microbiome") AND ("alpha diversity" OR Shannon OR Simpson OR Chao1 OR "species richness") AND (obes* OR overweight OR "body mass index" OR BMI) |
| **Web of Science Core Collection (original)** | Searched 10 February 2026; inception to search date | 8 | TS=("time-restricted eating" OR "time restricted eating" OR "time-restricted feeding" OR "time restricted feeding" OR TRE OR TRF) AND TS=("gut microbiota" OR "gut microbiome" OR "intestinal microbiome") AND TS=("alpha diversity" OR Shannon OR Simpson OR Chao1 OR "species richness") AND TS=(obes* OR overweight OR "body mass index" OR BMI); refined to Article. |
| **Embase via Ovid (original)** | Searched 10 February 2026; inception to search date | 94 | 1 'time restricted eating'/exp OR 'time restricted feeding'/exp; 2 (time-restricted NEAR/2 (eating OR feeding OR diet*)):ti,ab,kw; 3 (TRE OR TRF):ti,ab; 4 1 OR 2 OR 3; 5 'gastrointestinal microbiome'/exp OR 'gut microbiota'/exp; 6 (gut NEAR/2 (microbiom* OR microbiot*)):ti,ab,kw; 7 5 OR 6; 8 'alpha diversity'/exp OR (alpha NEAR/1 diversity):ti,ab,kw; 9 (Shannon OR Simpson OR Chao1 OR 'species richness'):ti,ab,kw; 10 8 OR 9; 11 'obesity'/exp OR 'overweight'/exp OR (obes* OR overweight):ti,ab,kw; 12 4 AND 7 AND 10 AND 11; 13 exclude conference abstract/paper; 14 exclude animal-only. |
| **Cochrane CENTRAL (original)** | Searched 10 February 2026; inception to search date | 22 | #1 MeSH [Time-Restricted Feeding]; #2 (time-restricted NEAR/2 (eating OR feeding)):ti,ab,kw; #3 (TRE OR TRF):ti,ab; #4 #1 OR #2 OR #3; #5 MeSH [Gastrointestinal Microbiome]; #6 (gut NEAR/2 (microbiom* OR microbiot*)):ti,ab,kw; #7 #5 OR #6; #8 (alpha NEAR/1 diversity):ti,ab,kw OR (Shannon OR Simpson OR Chao1 OR species richness):ti,ab; #9 MeSH [Obesity] OR MeSH [Overweight]; #10 (obes* OR overweight):ti,ab; #11 #9 OR #10; #12 #4 AND #7 AND #8 AND #11 |
| **PubMed (update)** | Searched 4 August 2026; 11 February–4 August 2026 | 9 | (("time-restricted eating"[Title/Abstract] OR "time restricted eating"[Title/Abstract] OR "time-restricted feeding"[Title/Abstract] OR "time restricted feeding"[Title/Abstract] OR TRE[Title/Abstract] OR TRF[Title/Abstract]) AND ("gut microbiota"[Title/Abstract] OR "gut microbiome"[Title/Abstract] OR microbiota[Title/Abstract] OR microbiome[Title/Abstract]) AND (obes*[Title/Abstract] OR overweight[Title/Abstract] OR "body mass index"[Title/Abstract])) AND ("2026/02/11"[Date - Publication] : "2026/08/04"[Date - Publication]) |
| **Europe PMC (update)** | Searched 4 August 2026; 11 February–4 August 2026 | 8 | ((TITLE_ABS:"time-restricted eating" OR TITLE_ABS:"time restricted eating" OR TITLE_ABS:"time-restricted feeding" OR TITLE_ABS:"time restricted feeding") AND (TITLE_ABS:"gut microbiota" OR TITLE_ABS:"gut microbiome" OR TITLE_ABS:microbiota OR TITLE_ABS:microbiome) AND (TITLE_ABS:obes* OR TITLE_ABS:overweight OR TITLE_ABS:"body mass index")) AND FIRST_PDATE:[2026-02-11 TO 2026-08-04] |
| **OpenAlex (update)** | Searched 4 August 2026; 11 February–4 August 2026 | 140 | Search phrase: "time-restricted eating" gut microbiome obesity; publication date filter 2026-02-11 to 2026-08-04. |
| **ClinicalTrials.gov (update)** | Searched 4 August 2026; 11 February–4 August 2026 | 26 | ("time-restricted eating" OR "time restricted eating" OR "time-restricted feeding") AND (microbiome OR microbiota) AND (obesity OR overweight) |

Original searches: 300 records; 184 unique records after removal of 116 duplicates.

Update searches: 183 records; 167 unique records after removal of 16 duplicates.

Overall: 483 records; 132 duplicates removed; 351 unique records screened.
