# Supplementary material for: Time-restricted eating and gut microbiota alpha diversity in adults with overweight or obesity: an updated rapid systematic review
Source: Front Nutr. 2026 Sep 9;13:1910444. doi: 10.3389/fnut.2026.1910444 (PMC13597893; doi:10.3389/fnut.2026.1910444)
Supplement: Supplementary file 2 [file Table_2.docx]

# Supplementary Table S2. Sample Reconciliation

Randomized or recruited, microbiome analytic, and paired samples are reported separately.

| **Study** | **Randomized / recruited N** | **Microbiome analytic N** | **Paired N** | **Reconciliation note** |
| --- | --- | --- | --- | --- |
| **Gabel 2020** | 14 | 14 | 14 | Uncontrolled pilot; all 14 contributed longitudinal microbiome data |
| **Mesnage 2022** | 50 | 49 | 49 | One recruited participant absent from the analytic comparison |
| **Wang 2023** | 28 | 26 | 26 | One TRE participant lacked baseline stool; 26 paired participants |
| **Liu 2024** | 69 | 51 | 51 | Selected TRE subgroup with stool; not a randomized microbiome contrast |
| **Li 2024** | 96 | 87 | 87 | 88 completed; paired sequencing succeeded for 87; TRE contrast 43 vs 44 |
| **Johnson 2025** | 20 | 16 | 5 | 21 samples from 16 participants; five had paired stools |
| **Habe 2025** | 197 | 76 | 76 | Early TRE+ER 33; late TRE+ER 23; ER 20 |
| **Zhang 2026** | 60 | 60 | 60 | Three-arm trial; arm-specific numerical data incompletely reported |
| **Dote-Montero 2026** | 197 | 138 | 138 | 182 provided stool; 138 had analyzable paired data |
| **Total** | 731 | 517 | 506 | The review denominator is the microbiome analytic total, not parent-trial enrollment. |
