# Supplementary material for: Time-restricted eating and gut microbiota alpha diversity in adults with overweight or obesity: an updated rapid systematic review
Source: Front Nutr. 2026 Sep 9;13:1910444. doi: 10.3389/fnut.2026.1910444 (PMC13597893; doi:10.3389/fnut.2026.1910444)
Supplement: Supplementary file 3 [file Table_3.docx]

# Supplementary Table S3. Tool-Specific Risk-of-Bias Assessment

The three instruments are reported separately because they assess different designs and do not share a common domain scale.

## A. RoB 2: randomized comparative studies

| **Study** | **D1** | **D2** | **D3** | **D4** | **D5** | **Overall** |
| --- | --- | --- | --- | --- | --- | --- |
| **Li** | Low | Low | Low | Low | Some concerns | Some concerns |
| **Johnson** | Low | Low | High | Low | Some concerns | High |
| **Habe** | Low | Low | Some concerns | Low | Some concerns | Some concerns |
| **Zhang** | Low | Low | Low | Low | Some concerns | Some concerns |
| **Dote-Montero** | Low | Low | Some concerns | Low | Some concerns | Some concerns |

RoB 2 domains: D1, randomization process; D2, deviations from intended interventions; D3, missing outcome data; D4, outcome measurement; D5, selection of the reported result.

| **Study** | **Basis for judgment** |
| --- | --- |
| **Li** | Microbiome was secondary and alpha-diversity analysis/reporting was not fully prespecified |
| **Johnson** | Only five of sixteen microbiome participants supplied paired samples |
| **Habe** | Secondary outcome with attrition at follow-up; end-of-intervention analysis retained separate arms |
| **Zhang** | Alpha-diversity numerical estimates and full contrast reporting were incomplete |
| **Dote-Montero** | Microbiome was secondary; 138 of 197 randomized participants had analyzable paired microbiome data |

## B. ROBINS-I: non-randomized controlled studies

| **Study** | **Confounding** | **Selection** | **Other domains** | **Reported result** | **Overall** |
| --- | --- | --- | --- | --- | --- |
| **Mesnage** | Serious | Moderate | Classification: Low; deviations: Low; missing data: Low; measurement: Low | Moderate | Serious |
| **Wang** | Serious | Moderate | Classification: Low; deviations: Moderate; missing data: Moderate; measurement: Low | Moderate | Serious |

| **Study** | **Basis for judgment** |
| --- | --- |
| **Mesnage** | Preference allocation and residual lifestyle confounding |
| **Wang** | Preference allocation, baseline imbalance, and imputation |

## C. JBI quasi-experimental checklist: uncontrolled studies

| **Study** | **Design** | **Key appraisal limitation** |
| --- | --- | --- |
| **Gabel** | Uncontrolled prospective pre-post | No concurrent control; small pilot sample; microbiome endpoint not powered |
| **Liu** | Uncontrolled longitudinal subgroup | Selected TRE subgroup; no randomized microbiome comparison; incomplete stool sample |

JBI checklist findings are summarized without converting them into RoB 2 or ROBINS-I domain ratings.
