# Supplementary material for: Time-restricted eating and gut microbiota alpha diversity in adults with overweight or obesity: an updated rapid systematic review
Source: Front Nutr. 2026 Sep 9;13:1910444. doi: 10.3389/fnut.2026.1910444 (PMC13597893; doi:10.3389/fnut.2026.1910444)
Supplement: Supplementary file 4 [file Table_4.docx]

# Supplementary Table S4. GRADE Certainty of Evidence

Certainty was rated as high, moderate, low, or very low for each defined evidence body using the GRADE domains. No pooled effect was estimated.

| **Evidence body** | **Studies (participants)** | **Risk of bias** | **Other GRADE domains** | **Publication bias** | **Certainty** |
| --- | --- | --- | --- | --- | --- |
| **Randomized comparative evidence** | 5 (377) | Serious | Inconsistency: Serious; indirectness: Not serious; imprecision: Serious | Suspected | Very low |
| **Non-randomized or uncontrolled evidence** | 4 (140) | Very serious | Inconsistency: Not serious; indirectness: Serious; imprecision: Serious | Suspected | Very low |
| **Overall alpha-diversity evidence** | 9 (517) | Serious | Inconsistency: Serious; indirectness: Not serious; imprecision: Serious | Suspected | Very low |

## Explanations

**Randomized comparative evidence:** Secondary and incompletely reported microbiome outcomes, discordant timing-specific findings, small effective samples, and no estimable common effect

**Non-randomized or uncontrolled evidence:** Preference allocation or no comparator, selected subgroups, heterogeneous co-interventions, small samples, and incomplete estimates

**Overall alpha-diversity evidence:** The direction and magnitude of TRE effects remain uncertain; failure to reach statistical significance is not evidence of no effect
